# Retinoic Acid-Regulated Epigenetic Marks Identify *Alx1* as a Direct Target Gene Required for Optic Cup Formation

**DOI:** 10.3390/genes16091071

**Published:** 2025-09-11

**Authors:** Marie Berenguer, Gregg Duester

**Affiliations:** 1 Development, Aging, and Regeneration Program, Sanford Burnham Prebys Medical Discovery Institute, La Jolla, CA 92037, USA; 2 Department of Medicine, Alice L. Walton School of Medicine, Bentonville, AR 72712, USA

**Keywords:** optic cup formation, retinoic acid signaling, RARE, H3K27ac, *Rdh10* knockout, *Alx1*

## Abstract

Background/Objectives: Retinoic acid (RA) is a transcriptional control agent that regulates several aspects of eye development including invagination of the optic vesicle to form the optic cup, although a target gene for this role has not been previously identified. As loss of RA synthesis in *Rdh10* knockout embryos affects the expression levels of thousands of genes, a different approach is needed to identify genes that are directly regulated by RA. Methods: Here, we combined ChIP-seq for the H3K27ac epigenetic mark with RNA-seq on optic field tissue from E10 wild-type and *Rdh10*−/− embryos that exhibit failure in optic cup formation. Results: We identified a small number of genes with decreased expression when RA is absent that also have a decreased presence of a nearby epigenetic gene activation mark (H3K27ac). One such gene was *Alx1* that also has an RA response element (RARE) located near the RA-regulated H3K27ac mark, providing evidence that RA directly activates *Alx1*. In situ hybridization studies showed that *Rdh10*−/− embryos exhibit a large decrease of *Alx1* expression in the optic field. CRISPR/Cas9 knockout of *Alx1* resulted in a defect in optic cup formation due to a failure of perioptic mesenchyme to migrate and separate the optic cup epithelium from the forebrain neuroepithelium. Conclusions: Our studies support a model in which RA functions to directly activate *Alx1* in perioptic mesenchyme to stimulate an early stage of eye development during which the optic vesicle folds into an optic cup that forms the retina.

## 1. Introduction

Retinoic acid (RA), derived from vitamin A (retinol), controls critical functions during eye development in humans, mice, and zebrafish [1,2,3,4,5]. Human studies have associated mutations in four components of the RA signaling pathway (*RBP4*, *STRA6*, *ALDH1A3*, *RARB*) and two RA target genes (*PITX2*, *FOXC1*) with anophthalmia or microphthalmia defects that occur during late eye development [1,2,3,4,5,6]. However, additional RA target genes may exist, particulaly for early eye development when optic cup formation occurs from E9.5–E10.5 in mouse embryos. RA controls the transcription of key genes by regulating the activity of RA receptors (RARs) that are bound to RA response elements (RAREs) [7,8]. Binding of RA to RARs that are bound to RAREs alters the recruitment of nuclear receptor coactivators (NCOAs) known to activate transcription, thus showing that RA activates transcription through RARE enhancers [7,8].

RA is synthesized from retinol in developing optic field tissues by the sequential activities of retinol dehydrogenase-10 (RDH10) [9] and all three retinaldehyde dehydrogenases (ALDH1A1, ALDH1A2, and ALDH1A3) [10,11]. In mouse embryos, *Rdh10* is expressed at E9.5–E10.5 in the optic vesicle and optic cup during the time when optic cup formation occurs [9,12]. At E10.5–E12.5, *Rdh10* knockout embryos exhibit misshapen optic cups with a shortened ventral retina that does not undergo normal separation from the forebrain neural epithelium; before folding, the eye is simply an optic vesicle outpocketing from the forebrain [9,13]. *Aldh1a1* (*Raldh1*) is expressed in the dorsal retina from E9.5 onwards, *Aldh1a2* (*Raldh2*) is expressed in the optic mesenchyme from E8.75–E9.5, and *Aldh1a3* (*Raldh3*) is expressed in the ventral retina from E8.5 onwards [11]. RA is required for normal folding of the optic vesicle to form the optic cup and ventral retina as shown in *Rdh10*−/− embryos [9] and in *Aldh1a1;Aldh1a2;Aldh1a3* triple knockouts [11]. *Aldh1a1;Aldh1a3* double knockouts form the optic cup (due to *Aldh1a2* RA synthesis), but at later stages they exhibit excessive neural crest-derived perioptic mesenchyme growth and anterior segment defects; in addition, *Pitx2* and *Foxc1* were found to be down-regulated in perioptic mesenchyme [11,14]. Studies on *Aldh1a1;Aldh1a3* double knockouts demonstrated that RA synthesized in the optic cup at E12.5 diffuses to the surrounding perioptic mesenchyme to activate *Pitx2* and *Foxc1* expression that regulates the migration of neural crest-derived perioptic mesenchyme, which contributes to cornea/eyelid morphogenesis and helps shape the overall optic cup morphology [11,14]. *Pitx2* and *Foxc1* knockouts exhibit anterior segment eye defects that arise after optic cup formation, but they still generate the optic cup [15,16]. Thus, RA likely regulates at least one additional gene needed at an early stage for optic cup formation.

We previously provided evidence that *Pitx2* has a nearby RARE and may thus be a direct RA target gene important for late aspects of eye formation [17]. However, there has been no success in identifying any RA target gene for optic cup formation. Identification of direct RA target genes is difficult as thousands of RAREs are observed in the mouse and human genomes [18,19]; in addition, the expression of thousands of genes is altered when RA is lost or added [20,21]. In order to fully understand eye RA signaling, it is essential to identify direct RA target genes that are activated in the developing eye.

As epigenetic studies have shown that histone H3 K27 acetylation (H3K27ac) is associated with gene activation [22], we propose that genes possessing nearby H3K27ac marks that are decreased by loss of RA may point to direct transcriptional targets of RA. Here, we performed genomic ChIP-seq for H3K27ac and RNA-seq studies on E10 mouse eye tissue from wild-type and *Rdh10*−/− mouse embryos lacking RA synthesis to globally identify direct RA target genes for the embryonic eye. Candidate target genes were defined as those with decreased mRNA levels that also have a nearby H3K27ac epigenetic mark that is decreased. This approach identified *Alx1* as a new candidate direct RA target gene, and CRISPR/Cas9 knockout studies demonstrated that *Alx1* is required for normal optic cup formation.

## 2. Materials and Methods

### 2.1. Ethics Statement

All mouse studies conformed to the regulatory standards adopted by the Institutional Animal Care and Use Committee at the SBP Medical Discovery Institute, which approved this study under Animal Welfare Assurance Number A3053-01 (approval #24-080, 20 September 2024). Animal care and use protocols adhered to the guidelines established by the National Institutes of Health (Stapleton, NY, USA).

### 2.2. Generation of Mutant Mouse Embryos

*Rdh10*−/− mice have been previously described [23]. E10 *Rdh10*−/− embryos were generated via timed matings of heterozygous parents; genotyping was performed by PCR analysis of yolk sac DNA. E10 eye tissue from wild-type and *Rdh10*−/− embryos was released from the head of the embryo by dissecting the frontal region containing both optic vesicles and frontonasal region. For in situ hybridization, we analyzed three wild-type and three *Rdh10*−/− embryos (*n* = 3 biological replicates). For RNA-seq studies, we analyzed three pools each of three wild-type and three *Rdh10*−/− eye tissues (*n* = 3 biological replicates). For ChIP-seq, we analyzed two pools each of 60 wild-type and 60 *Rdh10*−/− eye tissues (*n* = 2 biological replicates). For *Alx1* CRISPR mutants, we analyzed three wild-type and three mutant embryos (*n* = 3 biological replicates). The mouse strain used was an outbred mix of Black Swiss and C57BL/6. For all mouse studies, no animals were excluded.

### 2.3. RNA-Seq Analysis

Total RNA was extracted from craniofacial/eye tissues of E10 wild-type and *Rdh10*−/− embryos (9 each). RNA libraries were prepared using the NEB Biolabs Next Ultra II Directional RNA Library Prep Kit with the PolyA selection module (New England Biolabs, Ipswich, MA, USA #E7490). Sequencing was performed on a NovaSeq platform generating 40 million reads per sample with single read lengths of 75 bp, generating 25MM PE50 reads per sample. Sequences were aligned to the mouse mm10 reference genome using TopHat splice-aware aligner; transcript abundance was calculated using the Expectation–Maximization approach; fragments per kilobase of transcript per million mapped reads (FPKM) was used for sample normalization; the Generalized Linear Model likelihood ratio test in edgeR software (version 4) was used as a differential test. High throughput DNA sequencing was performed in the La Jolla Immunology Genomics Core. The RNA-seq data is available at Gene Expression Omnibus (GEO) under GSE297809.

### 2.4. Chromatin Immunoprecipitation (ChIP) Sample Preparation for ChIP-Seq

For ChIP-seq, we used craniofacial/eye tissue from E10 wild-type and *Rdh10*−/− embryos (60 each) dissected in modified PBS, i.e., phosphate-buffered saline containing 1X complete protease inhibitors (concentration recommended for the use of soluble EDTA-free tablets sold by Roche, Basel, Switzerland #11873580001) and 10 mM sodium butyrate as a histone deacetylase inhibitor (Sigma, Tokyo, Japan # B5887). Samples were processed similar to previous methods [23]. Dissected eye tissues were briefly centrifuged in 1.5 mL tubes and excess PBS dissection buffer was removed. For cross-linking of chromatin DNA and proteins, 500 μL 1% formaldehyde was added and the eye samples were minced by pipetting up and down with a 200 μL pipette tip and then incubated at room temperature for 15 min. To stop the cross-linking reaction, 55 μL of 1.25 M glycine was added and samples were rocked at room temperature for 5 min. Samples were centrifuged at 5000 rpm for 5 min and the supernatant was carefully removed and discarded. A wash was performed in which 1000 μL of ice-cold modified PBS was added and mixed by vortexing followed by centrifugation at 5000 rpm for 5 min and careful removal of the supernatant, which was discarded. This wash was repeated. Cross-linked eye samples were stored at −80 C until all samples were collected.

Chromatin was fragmented by sonication. Cross-linked eye samples were pooled, briefly centrifuged, and excess PBS removed. Then, 490 μL lysis buffer (modified PBS containing 1% SDS, 10 mM EDTA, 50 mM Tris-HCl, pH 8.0) was added, mixed by vortexing, and then the samples were incubated on ice for 10 min. Samples were divided into four sonication microtubes; Covaris AFA Fiber Pre-Slit Snap-Cap 6, 16 mm (Covaris, LLC; Woburn, MA, USA #520045) with 120 μL per tube. Sonication was performed with a Covaris Sonicator with the following settings: Duty, 5%; Cycle, 200; Intensity, 4; #Cycles, 10; 60 s each for a total sonication time of 14 min. The contents of the four tubes were re-combined by transfer to a single 1.5 mL microtube, which was then centrifuged for 10 min at 13,000 rpm and the supernatant was transferred to a fresh 1.5 mL microtube. These conditions were found to shear eye tissue DNA to an average size of 300 bp using a 5 μL sample for Bioanalyzer analysis. At this point, 20 μL was removed for each sample (wild-type eye tissue and also *Rdh10*−/− eye tissue) and stored at −20 °C to serve as input DNA for ChIP-seq.

Each sample was divided into four 100 μL aliquots to perform immunoprecipitation with ChIP-grade antibodies for H3K27ac (Active Motif, Carlsbad, CA, USA Cat#39133) in duplicate. Immunoprecipitation was performed using the Pierce Magnetic ChIP Kit (Thermo Scientific, Dreieich, Germany #26157) following the manufacturer’s instructions. The immunoprecipitated samples and input samples were subjected to reversal of cross-linking by adding water to 500 μL and 20 μL 5 M NaCl, vortexing, and incubation at 65 C for 4 h; then addition of 2.6 μL RNase (10 mg/mL), vortexing, and incubation at 37 C for 30 min; then addition of 10 μL 0.5 M EDTA, 20 μL 1 M Tris-HCl, pH 8.0, 2 μL proteinase K (10 mg/mL; Thermo Fisher Scientific, Waltham, MA, USA #EO0491), vortexing, and incubation at 45 C for 1 h. DNA was extracted using ChIP DNA Clean & Concentrator (Zymo, Irvine, CA, USA # D5201 & D5205). After elution from the column in 50 μL of elution buffer, the DNA concentration was determined using 2 μL samples for Bioanalyzer analysis. The two input samples ranged from 16–20 ng/μL and the immunoprecipitated samples ranged from 0.1–0.2 ng/μL (5–10 ng per 60 pooled eye tissues). For ChIP-seq, 2 ng was used per sample to prepare libraries for DNA sequencing.

### 2.5. ChIP-Seq Genomic Sequencing and Bioinformatic Analysis

Libraries for DNA sequencing were prepared according to the instructions accompanying the NEBNext DNA Ultra II kit (catalog # E7645S; New England Biolabs, Inc., Ipswich, MA, USA). Libraries were sequenced on the NextSeq 500 following the manufacturer’s protocols, generating 40 million reads per sample with single read lengths of 75 bp. Adapter remnants of sequencing reads were removed using cutadapt v1.18 [24]. ChIP-Seq sequencing reads were aligned using STAR aligner version 2.7 to Mouse genome version 38 [25]. Homer v4.10 [26] was used to call peaks from ChIP-Seq samples by comparing the ChIP samples with matching input samples. Homer v4.10 was used to annotate peaks to mouse genes and quantify read counts to peaks. The raw read counts for different peaks were compared using DESeq2 [27]. *p* values from DESeq2 were corrected using the Benjamini & Hochberg (BH) method for multiple testing errors [28]. Peaks with BH corrected *p* value < 0.05 (BHP < 0.05) were selected as significantly differentially marked peaks. Transcription factor binding sites motif enrichment analyses were performed using Homer v4.10 [26] to analyze the significant RA-regulated ChIP-seq peaks for RARE sequences; DR1 RAREs related to the TR4 (NR),DR1 motif; DR2 RAREs related to Reverb (NR), DR2; and DR5 RAREs related to RAR:RXR (NR), DR5. High throughput DNA sequencing was performed in the Sanford Burnham Prebys Genomics Core and bioinformatics analysis was performed in the Sanford Burnham Prebys Bioinformatics Core. The ChIP-seq data is available at GEO under GSE298400.

### 2.6. Generation of Mutant Embryos by CRISPR/Cas9 Mutagenesis

CRISPR/Cas9 gene editing was performed using Alt-R CRSIPR/Cas9 tracrRNA (Integrated DNA Technologies, Inc., Coralville, IA, USA) combined with two crRNA guides (generated by Integrated DNA Technologies, Inc.) that target *Alx1* exon 1 to generate frameshift null mutations. crRNAs were designed with maximum specificity using the tool at Integrated DNA Technologies, Inc. to ensure that each crRNA had no more than 17 out of 20 matches with any other site in the mouse genome and that those sites are not located within exons of other genes. The guides used here for targeting the mouse *Alx1* gene were as follows: TTTTACGGCAAAGCGACGGC and AGCATCACGTGCGCCTGGAC.

Injection of mouse fertilized eggs and generation of *Alx1* CRISPR embryos was performed as follows. For injection into mouse embryos, a solution containing 1.2 mM Cas9 protein (Integrated DNA Technologies, Inc.), AltR nuclease (Integrated DNA Technologies, Inc.), 25 mM in HEPES, and 0.6 mM for each crRNA/tracrRNA duplex used was prepared in nuclease free water. Fertilized oocytes were collected from 3–4 week-old superovulated C57Bl6 females prepared by injecting 5 IU each of pregnant mare serum gonadotrophin (PMSG) (Sigma Aldrich, Schnelldorf, Germany) and human chorionic gonadotropin (hCG) (Sigma Aldrich). Fertilized oocytes were then transferred into M2 medium (Millipore, Darmstadt, Germany) and injected with the Cas9 mRNA/tracrRNA/crRNA solution into the cytoplasm. Injected embryos were implanted into recipient pseudo-pregnant ICR female mice. Implanted females were sacrificed to obtain F0 E10.5 embryos. For genotyping, yolk sac DNA was collected and PCR products were generated using primers flanking the crRNA target sites; PCR products were subjected to DNA sequence analysis from both directions using either an upstream primer (CGAGAAGTTTGCCCTGAAGA) or a downstream primer (GTACCTCACTTCGGGGAAGG). For each gene analyzed, embryos were classified as homozygous if only frame-shift alleles were detected but no wild-type sequence. An example of a frame-shift mutation observed in *Alx1* exon 1 is as follows:

WT: CAAATGCGTGCAGGCCTTCGGGCC
KO: CAAATGCGTGCAGCCTTCGGGCC

### 2.7. In Situ Hybridization Gene Expression Analysis

E10.5 embryos were fixed in paraformaldehyde at 4 °C overnight, dehydrated in methanol, and stored at −20 °C. Detection of mRNA was performed by whole mount in situ hybridization as previously described [29].

### 2.8. Analysis of Eye Morphology

Histological examination of embryonic eyes was performed on paraffin embedded and sectioned tissues stained with hematoxylin/eosin as previously described [11].

## 3. Results

### 3.1. Comparison of H3K27ac ChIP-Seq and RNA-Seq for Rdh10−/− Eye Tissue

As direct RA target genes for optic cup formation are unknown, we used a combined RNA-seq and ChIP-seq approach that we described previously [23] to globally identify direct RA target genes for eye development; i.e., genes that when RA is missing exhibit a significant decrease in expression (analyzed by RNA-seq) and also a significant decrease in H3K27ac, an epigenetic mark associated with gene activation (analyzed by ChIP-seq).

Embryonic eye tissues were obtained from E10 embryos by dissecting just the frontal region of the head. As the developing eye tissue is very small at E10, the samples contained the optic vesicles and other nearby craniofacial tissues that comprise the face plus some forebrain adjacent to where the optic vesicles emerge as an out-pocketing of the forebrain; we reasoned that our knockout studies of an RA target gene identified from this tissue would be able to identify a gene required for eye development as opposed to development of other craniofacial tissues. We performed RNA-seq analysis comparing E10 eye tissue from wild-type embryos and *Rdh10*−/− embryos that lack the ability to produce RA in the entire head, including the optic vesicles and surrounding mesenchyme [30]. This analysis identified genes whose mRNA levels in eye tissue were significantly decreased (359 genes) or increased (1040 genes) when RA was absent (FPKM > 0.5; a cut-off of log_2_ <−0.85 or >0.85 was employed; RNA-seq data available at Gene Expression Omnibus (GEO) under accession number GSE297809).

We performed ChIP-seq analysis for the H3K27ac epigenetic mark comparing E10 eye tissue from wild-type and *Rdh10*−/− embryos isolated as described above. This analysis identified RA-regulated H3K27ac ChIP-seq peaks that were either decreased (located within or near 3340 genes) or increased (located within or near 3357 genes) using a log_2_ cut-off of <−0.51 or >0.51; the ChIP-seq data is available at GEO under accession number GSE298400.

**Figure 1:**
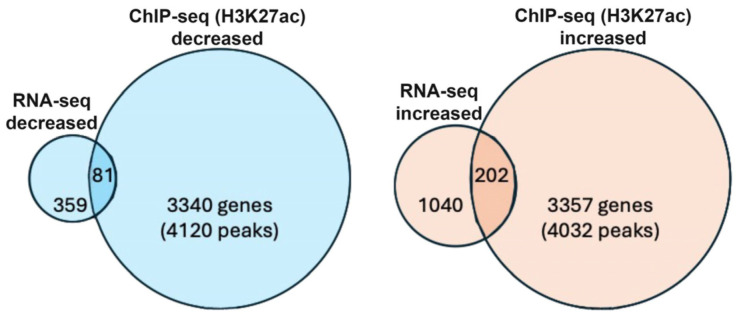
Bioinformatic analysis of genes identified as early eye direct RA target genes. Venn diagram showing the number of genes that have both RA-regulated expression (decreased or increased) and RA-regulated deposition of nearby H3K27ac marks (decreased or increased) following loss of RA. Individual values for H3K27ac (wild type vs. *Rdh10*−/−) can be found in ChIP-seq data deposited at GEO under accession number GSE298400. Individual values for RNA-seq (wild type vs. *Rdh10*−/−) can be found in the data deposited at GEO under accession number GSE297809.

In order to identify genes that are good candidates for being transcriptionally activated or repressed by RA (RA target genes), we compared our RNA-seq and H3K27ac ChIP-seq results to identify RA-regulated ChIP-seq peaks where nearby genes have significant changes in expression in wild-type vs. *Rdh10*−/− based on RNA-seq. We found 81 genes that exhibited both decreased expression and decreased peaks for H3K27ac, plus 202 genes that exhibited increased expression and increased peaks for H3K27ac (Figure 1).

We focused upon the 81 candidate RA target genes that exhibited both decreased expression and decreased H3K27ac deposition. Our combined RNA-seq and H3K27ac ChIP-seq results for this group appear to be high quality: they include several genes that were already known to be part of the optic vesicle RA regulatory pathway; i.e., *Rarb* (nuclear RA receptor), *Rdh10* (first step of RA synthesis—conversion of retinol to retinaldehyde), and *Aldh1a3* (second step of RA synthesis—conversion of retinaldehyde to RA). These three genes are also included within a group of 12 genes with the greatest decreases in expression observed by RNA-seq, and here we show their corresponding changes in H3K27ac deposition (Table 1). Among these genes, seven exhibit decreased H3K27ac including *Rarb*, *Rdh10*, and *Aldh1a3*. This group includes *Alx1*, which has a large value for decreased H3K27ac deposition when RA is missing.

### 3.2. Alx1 Expression in Wild-Type vs. Rdh10 KO Embryos

**Figure 2:**
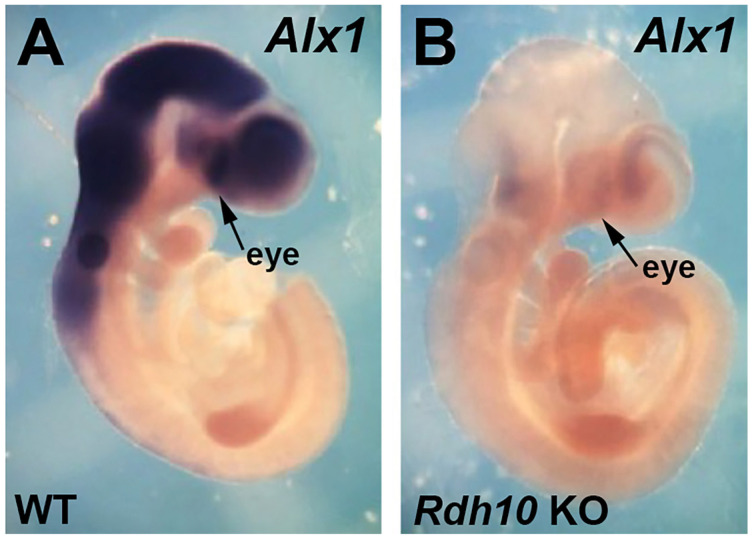
Comparison of *Alx1* expression in E10.5 wild-type vs. *Rdh10* KO. (**A**,**B**) Shown are mouse E10.5 embryos, either wild-type (WT) or *Rdh10*−/− (*Rdh10* KO), subjected to in situ hybridization to detect *Alx1* mRNA. The arrows point to the eye region where the optic cup is forming.

*Alx1* has previously been shown to be expressed in mouse perioptic and craniofacial mesenchyme surrounding the optic vesicle/cup at E9.5–E10.5, but not in the optic vesicle/cup itself, and then at later stages, *Alx1* is expressed in the cornea [31,32]. Here, we compared expression of *Alx1* in E10.5 wild-type and *Rdh10*−/− embryos. We found that loss of RA in *Rdh10*−/− embryos results in a very large decrease in *Alx1* expression in the eye region; *n* = 3 (Figure 2). These results corroborate the RNA-seq data, showing that *Alx1* expression is decreased in the *Rdh10* KO.

### 3.3. Identification of a RARE Associated with RA-Regulated Deposition of the H3K27ac Epigenetic Mark near Alx1

**Figure 3:**
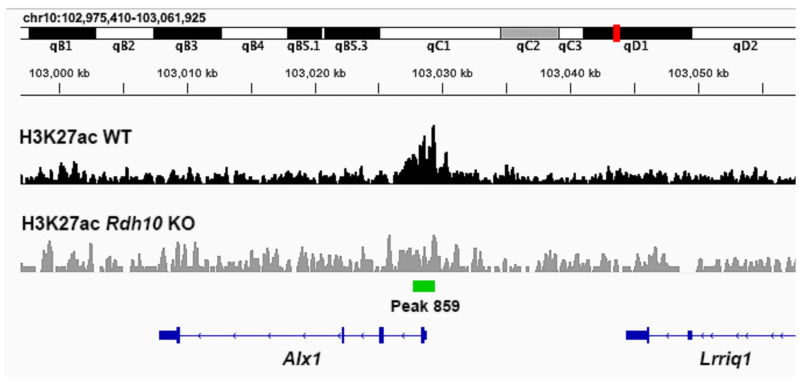
Chromosomal genomic DNA view showing comparison of H3K27ac ChIP-seq data near *Alx1* in wild-type vs. *Rdh10*−/− eye tissue. Shown here is the region along mouse chromosome 10 where *Alx1* resides with its transcription direction going from right to left. The ChIP-seq data for H3K27ac from E10.5 eye tissue, either wild-type (WT) or *Rdh10*−/− (*Rdh10* KO), is plotted under this chromosomal region. Observed is peak 859 overlapping the transcription start site for *Alx1* that is significantly decreased in the *Rdh10* K0.

A genomic comparison of the H3K27ac ChIP-seq data near the 5′ end of *Alx1* for wild-type and *Rdh10*−/− eye tissue is shown here (Figure 3). In wild-type tissue, a significant peak of H3K27ac deposition is observed overlapping the transcription start site of *Alx1*, and this peak is reduced in the *Rdh10* KO.

**Figure 4:**
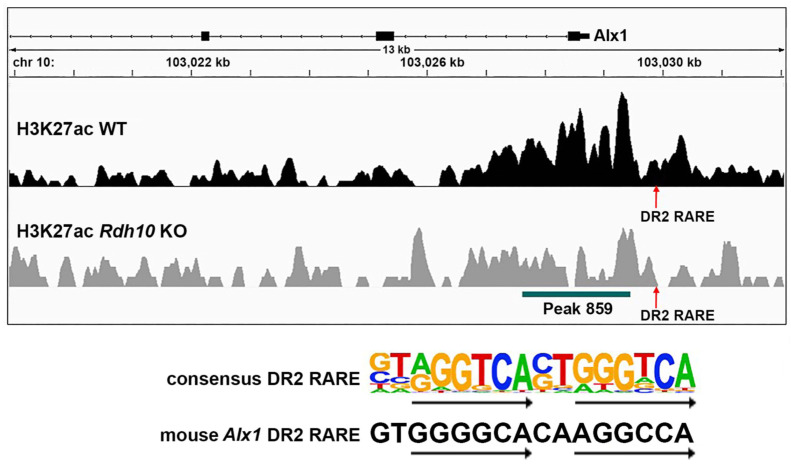
RARE near the RA-regulated H3K27ac peak located upstream of the *Alx1* transcription start site. Shown is a mouse chromosome 10 view of the H3K27ac ChIP-seq peaks for wild-type (WT) and *Rdh10*−/− (*Rdh10* KO) from E10.5 eye tissue showing a RA response element (RARE) just upstream of peak 859 that overlaps the *Alx1* transcription start site. This is a DR2 RARE, which consists of two 6 bp direct repeats separated by a 2 bp spacer as shown by the consensus DR2 RARE sequence. The DR2 RARE upstream of *Alx1* matches the consensus in 10 of the 12 bp that comprise the two 6 bp repeats, and it matches 1 bp from the 2 bp spacer.

As RA target genes need to be associated with a RARE, the DNA sequences within and near the RA-regulated H3K27ac ChIP-seq peak for *Alx1* were analyzed for the presence of RARE DNA sequences (i.e., direct repeats with spacers of either 1, 2, or 5 bp; DR1, DR2, or DR3) using methods we previously described [23]. We found a DR2 RARE approximately 500 bp upstream of the H3K27ac peak overlapping the *Alx1* transcription start site (Figure 4). These results provide evidence that *Alx1* may be directly regulated by RA during optic cup formation.

### 3.4. Alx1 Is Required for Normal Optic Cup Formation

**Figure 5:**
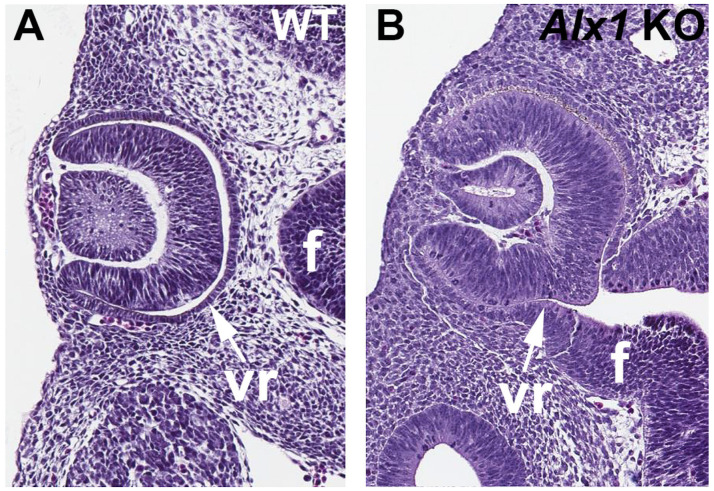
Eye sections comparing wild-type vs. *Alx1* CRISPR KO embryos showing the optic cup defect. (**A**,**B**) Comparison of E10.5 wild-type (WT) and *Alx1* knockout (KO) eyes showing a failure of the optic cup to fold properly in the *Alx1* CRISPR embryos, leaving the ventral retina (vr) still connected to the forebrain neural epithelium (f).

In order to test whether *Alx1* is required for normal optic cup formation, we used CRISPR/Cas9 technology to generate *Alx1* knockout embryos that contain a deletion in exon 1 that introduces a frameshift mutation. Wild-type and *Alx1* CRISPR embryos collected at E10.5 were sectioned in the dorsoventral plane through the eyes. The sections revealed that *Alx1* KO eyes display a failure of the ventral optic cup (future ventral retina) to fold properly; *n* = 3 mutants compared to two WT (Figure 5). In the *Alx1* CRISPR embryos, the optic vesicle did not completely fold to generate a normal optic cup with a prospective retinal epithelium that separates from the nearby forebrain epithelium as seen in wild type embryos.

## 4. Discussion

Previous studies have demonstrated that RA signaling is required for folding of the optic vesicle at E9.5 to form the optic cup at E10.5 as well as for further morphogenesis of the optic cup at later stages [9,11,13,14]. However, the identification of an RA target gene required for optic cup formation has not been described. Here, our RNA-seq findings combined with H3K27ac epigenetic ChIP-seq findings comparing wild-type to *Rdh10*−/− RA-deficient eye tissue has provided a means for identifying new candidate direct RA target genes that may be required for optic cup formation. By focusing on RA-regulated genes that also have a nearby RA-regulated H3K27ac epigenetic mark associated with a RARE, our approach can narrow down the list of RA-regulated genes to those that are direct targets of RA transcriptional control. Our method allows identification of genes that are likely to be transcriptional targets of the RA signaling pathway as opposed to genes whose expression is altered by effects downstream of RA signaling, such as changes in the expression of other transcription factors or possibly post-transcriptional effects on mRNA abundance that are picked up by RNA-seq analysis.

Here, in our studies on *Rdh10*−/− eye tissue, we were able to narrow down the hundreds of genes identified with RNA-seq that have significant reductions in gene expression to just a few candidate direct RA target genes that also have significant reductions in nearby H3K27ac deposition. From this combined RNA-seq and H3K27ac ChIP-seq comparison, *Alx1* appeared as a very good potential direct RA target gene. This view was further strengthened by analysis of *Alx1* expression in E10.5 embryos showing a large decrease in expression in *Rdh10*−/− compared to wild-type. This view was additionally supported by transcription factor binding site analysis that revealed a DR2 RARE near the RA-regulated H3K27ac peak overlapping the *Alx1* transcription start site. After demonstrating that *Alx1* knockout embryos fail to undergo normal optic cup formation, we conclude that *Alx1* may be a direct RA target gene whose expression is activated by RA in order to provide instructions for how the optic vesicle folds to form a normal optic cup. As *Alx1* has previously been shown to be expressed in perioptic mesenchyme surrounding the optic vesicle/cup [31,32], it is possible that *Alx1* controls the migration of cranial neural crest-derived perioptic mesenchyme that helps pattern the folding of the optic cup, similar to how *Pitx2* controls cranial neural crest perioptic mesenchyme for eye anterior segment morphogenesis at later stages of eye morphogenesis [15,16,33]. Previous knockout studies have shown that *Alx1* is required for normal craniofacial mesenchyme migration as it relates to frontonasal dysplasia [34]. Thus, we suggest here that RA generated in the optic vesicle controls the folding process that forms an optic cup by diffusing into surrounding perioptic mesenchyme where it activates the expression of *Alx1*, which controls perioptic mesenchyme function. This folding process generates the epithelium in the posterior portion of the optic cup that will later differentiate into the retina, thus separating the retinal epithelium from the forebrain neural epithelium. Future studies can address the mechanism through which RA controls *Alx1* expression and the mechanism underlying how *Alx1* controls perioptic mesenchyme function during optic cup formation.

Our studies reported here demonstrate the power of combining gene knockout mice, RNA-seq, and ChIP-seq for epigenetic marks to identify direct RA target genes required for a particular developmental process. Here, we have revealed that RA is required to activate expression of *Alx1*, which is then required in the process of optic cup formation. This knowledge is essential for understanding the mechanisms underlying how RA signaling controls eye development. This knowledge will also help determine how human eye defects occur, identify genes that may be mutational targets causing human eye defects, and improve strategies to treat eye defects.

Limitations of this study: The developing eyes (optic vesicles/cups) at E10 are quite small and therefore not possible to dissect free of surrounding tissues. Thus, as tissues for RNA-seq and ChIP-seq analyses were obtained by dissecting the frontal region of the head that contains the eyes along with other craniofacial tissues, the RNA-seq and ChIP-seq analyses would not necessarily detect RA target genes for only the developing eye. In this case, we relied on gene expression and knockout studies of the RA target gene identified from this tissue (*Alx1*) to identify it as a gene required for eye development.

## Figures and Tables

**Table 1:** Comparison of datasets for RNA-seq and ChIP-seq (H3K27ac) from E10 eye regions (*Rdh10* KO vs. wild type) to identify genes that have significant reductions in mRNA expression and significant changes in nearby deposition of H3K27ac when RA is missing.

Gene with Altered Expression in *Rdh10* KO	log_2_ Fold Change in Gene Expression: RNA-seq for *Rdh10* KO vs. WT	Nearby H3K27ac ChIP-seq RA-Regulated Peak for *Rdh10* KO vs. WT (mm10 Coordinates)	log_2_ Fold Change: H3K27ac ChIP-seq for *Rdh10* KO vs. WT
*Adck4*	−0.5	chr7: 27232464-27233503	1.04
*Hspa8*	−0.498	chr9: 40798968-40802290	−0.284
*Sdf2l1*	−0.414	chr16: 17131820-17133225	0.052
*Rad51c*	−0.401	chr11:87403988-87404734	0.206
*Car2*	−0.389	chr14: 70152974-70154100	−0.236
*Rarb*	−0.37	chr14: 16554360-16555273	−0.165
*Aldh1a3*	−0.367	chr7:66449969-66450726	−0.165
*Alx1*	−0.363	chr10:103027606-103029451	−0.879
*Cln5*	−0.344	chr14:103069656-103070583	0.341
*Rps14*	−0.342	chr18: 60774247-60775597	0.218
*Manf*	−0.342	chr9:106891507-106892882	−0.602
*Rdh10*	−0.341	chr1:16248679-16249396	−0.395

## Data Availability

The accession numbers of the sequence data that are reported in this study are listed in the legend for Figure 1.
